# Application of resting-state EEG theta /alpha power ratio analysis for diagnosing amnestic mild cognitive impairment

**DOI:** 10.1038/s41598-026-46950-8

**Published:** 2026-05-11

**Authors:** Yang Liang, Peixue Li, Ying Wang, Fangfang Jing, Chunbo Dong, Li Zhao

**Affiliations:** 1 Department of Neurology, The First Affiliated Hospital of Dalian Medical University, Dalian, 116011 China; 2 Department of Neuroelectrophysiology, Central Hospital of Dalian University of Technology (Dalian Municipal Central Hospital), Dalian, 110600 Liaoning China

**Keywords:** Amnestic mild cognitive impairment (aMCI), Electroencephalography (EEG), Relative spectral power, Diagnosis, Biomarker, Biomarkers, Medical research, Neurology, Neuroscience

## Abstract

This study aimed to investigate spectral power and the theta/alpha power ratio of resting-state electroencephalography (EEG) in patients with amyloid-positive amnestic mild cognitive impairment (aMCI) compared to normal controls (NC). Twenty-four patients with aMCI, confirmed by Pittsburgh Compound B positron emission tomography (PiB-PET), and twenty-four age-matched NC participants were included. Resting-state EEG recordings were analyzed for spectral power across delta, theta, alpha, and beta bands. Group comparisons and receiver operating characteristic (ROC) curve analyses, corrected for multiple comparisons using false discovery rate (FDR), were performed to assess diagnostic utility. Compared to NC, patients with aMCI exhibited EEG slowing, characterized by higher power in lower frequency bands and lower power in higher frequency bands. Regional analysis showed that the theta/alpha power ratio in the occipital region provided the best discrimination between groups (area under the curve [AUC] = 0.86), with high sensitivity and specificity (both > 91.7%). These findings indicate that patients with biomarker-confirmed aMCI present distinct, widespread electrophysiological alterations, possibly reflecting underlying neuropathological changes such as cholinergic dysfunction. This study provides a regional, quantitative evaluation of EEG spectral measures and their diagnostic performance in a PiB-PET-characterized aMCI cohort, offering exploratory evidence for the theta/alpha ratio as a potential adjunctive biomarker. Further validation in larger, independent samples is warranted.

## Introduction

Mild cognitive impairment (MCI) is characterized by an observable decline in memory or cognitive function, although individuals affected can still manage daily activities independently^1^. When memory impairment predominates, the condition is termed amnestic mild cognitive impairment (aMCI), which carries a higher risk of progressing to Alzheimer’s disease (AD)^2^. Studies indicate that 47%-75% of patients with aMCI have positive amyloid-beta (Aβ) deposition identified by PET, signifying an increased risk of developing AD^3^. Furthermore, 45%-82% of Aβ-positive aMCI patients progress to AD dementia within 2–3 years, compared to only 0%-11% of Aβ-negative patients^4^. The global prevalence of aMCI continues to rise annually, particularly among aging populations^5^. Patients with aMCI frequently experience considerable psychological stress and decreased quality of life^6^. Additionally, aMCI imposes a substantial economic burden on society and healthcare systems^7^. Thus, there is an urgent need for accessible, objective, and non-invasive tools to support the early identification of aMCI, particularly in the context of underlying AD pathology.

In current clinical practice, the diagnosis of aMCI primarily relies on biomarkers from cerebrospinal fluid (CSF), neuropsychological tests, neuroimaging, and neurophysiological assessments^8^. CSF biomarkers, including Aβ and tau proteins, are highly sensitive and specific for identifying AD pathology^9^. However, CSF collection requires invasive lumbar puncture, which may deter patient participation^10^. Neuroimaging biomarkers, such as amyloid-PET, are highly specific for detecting in vivo Aβ deposition^11^. Amyloid-PET positivity is a key criterion for defining “MCI due to AD”^12^. Although valuable, PET imaging is costly, requires advanced infrastructure, and involves radiation exposure. These factors limit its suitability for large-scale screening^13^. Neuropsychological tests, such as the Mini-Mental State Examination (MMSE) and Montreal Cognitive Assessment (MoCA), are widely used in clinical practice because of their practicality^14,15^. However, they may be time-consuming, require active patient cooperation, are clinician-dependent, and can be influenced by educational and cultural factors^16,17^.

Electroencephalography (EEG) is a non-invasive and cost-effective electrophysiological tool that directly records brain activity with high temporal resolution^18^. Quantitative EEG analysis has been widely applied to neurological disorders, including MCI and AD^19^. Compared with healthy older adults, individuals with MCI and AD often exhibit “EEG slowing,” characterized by increased power in slow-frequency bands (e.g., theta) and decreased power in faster bands (e.g., alpha)^20,21^. This spectral pattern is a well-established hallmark of prodromal and early AD^22^. Reductions in alpha power are of particular interest because alpha oscillations play a causal role in attentional processes^23^ and are linked to the locus of attentional focus^24^. They also support neural integration and segregation, which are cognitive functions frequently impaired in aMCI^25^. Among EEG metrics, the theta-to-alpha power ratio (θ/α ratio) has consistently demonstrated utility in distinguishing individuals with MCI from cognitively normal controls (NC)^26–28^. However, findings across specific frequency bands vary between studies due to cohort heterogeneity, diagnostic criteria, and analytical methods^29,30^.

Although the diagnostic value of EEG spectral measures, including the θ/α ratio, has been reported in MCI, many prior studies included diagnostically heterogeneous cohorts without rigorous biomarker confirmation of underlying AD pathology. Therefore, the present study aimed to evaluate the diagnostic performance of regional resting-state EEG spectral power and the theta/alpha power ratio in a well-characterized cohort of amyloid-PET-positive aMCI (i.e., MCI due to AD) compared with age-matched healthy controls. Regional analyses were performed across five predefined brain regions (frontal, central, temporal, parietal, and occipital), based on the standard 10–20 EEG electrode system and consistent with previous quantitative EEG (qEEG) studies in MCI and early AD. Receiver operating characteristic (ROC) analysis was conducted to assess the potential of these EEG metrics as accessible indicators for this specific at-risk population.

## Materials and methods

### Participants

Patients with aMCI were recruited consecutively from the outpatient neurology clinic of the First Affiliated Hospital of Dalian Medical University between 2022 and 2024. All participants fulfilled the core clinical criteria for “MCI due to AD,” as proposed by the National Institute on Aging-Alzheimer’s Association (NIA-AA) guidelines^31^. All aMCI patients were confirmed as Pittsburgh compound B PET (PiB-PET) positive, verifying the presence of AD pathology.

Inclusion criteria were: (a) subjective memory complaints corroborated by an informant; (b) objective episodic memory impairment, defined as a delayed recall score on the Rey Auditory Verbal Learning Test ≥ 1.5 standard deviations below norms adjusted for age and education; (c) preserved global cognitive function (Mini-Mental State Examination [MMSE] ≥ 24) and intact activities of daily living; and (d) a Clinical Dementia Rating (CDR) score of 0.5.

The sample size (*n* = 24 per group) was determined based on feasibility and the limited availability of PiB-PET-confirmed aMCI participants, a rare and well-characterized clinical population. Post-hoc power analysis confirmed adequate power (> 80%) to detect large effect sizes (Cohen’s d ≥ 0.8), consistent with the robust group differences observed. However, the small sample size may limit the stability of ROC estimates and the generalizability of findings; these constraints are explicitly addressed in the limitations section.

Additionally, 24 healthy NCs matched for gender, age, and education were recruited from the community. All participants were independently evaluated by at least two senior neurologists. Assessments included the MMSE, Montreal Cognitive Assessment (MoCA), Activities of Daily Living (ADL) scale, Hamilton Anxiety Rating Scale (HAMA), Hamilton Depression Rating Scale (HAMD), and Hachinski Ischemic Scale (HIS) to exclude vascular dementia^31–35^.

The study protocol involving human participants was reviewed and approved by the Medical Ethics Committee of the First Affiliated Hospital of Dalian Medical University. Written informed consent was obtained from all participants.

### EEG collection and preprocessing

EEG data were collected in a dedicated clinic room in the Department of Neurology at the First Affiliated Hospital of Dalian Medical University. Before recording, participants were asked about the intake of stimulants, including nicotine, caffeine, or alcohol, on the day of testing. Those who reported no consumption underwent standard scalp cleansing to improve electrode contact. Participants were seated comfortably and instructed to remain quiet and relaxed to minimize artifacts. Consistent with prior studies, only eyes-closed resting-state EEG data were analyzed. Recordings lasted 10 min. EEG signals were acquired using an amplifier (bandwidth: 0.1–80 Hz; sampling rate: 1000 Hz).

Signals were recorded from 21 electrodes placed according to the International 10–20 system, with linked earlobes (A1 + A2) used as reference electrodes^36^. Raw EEG signals were filtered using a band-pass filter (1–55 Hz) and a 50 Hz notch filter to remove power-line interference. Data were then re-referenced using the common average reference (CAR) method. Motion artifacts (e.g., eye blinks, muscle activity, electrode displacement) were visually inspected by two independent researchers using a 25-s sliding window. Epochs were excluded if artifact-contaminated segments exceeded 30% of the window length or if signal amplitude exceeded ± 100 µV. Disagreements were resolved by consensus. This process yielded an average of 6.2 min of artifact-free EEG data per participant. Continuous clean data were segmented into non-overlapping 5-s epochs. For regional analysis, electrodes were grouped into five regions based on anatomical proximity and established conventions: frontal (FP1, FP2, F3, Fz, F4), central (C3, Cz, C4), temporal (T3, T4, T5, T6, F7, F8), parietal (P3, Pz, P4), and occipital (O1, O2)^36^.

### Spectral power analysis

Spectral analysis was performed on 5-s epochs. Data were first down-sampled to 250 Hz to reduce computational demand, yielding 1250 data points per epoch. Power spectral density was estimated using the Welch method. A Hamming window of 125 points (0.5 s) was applied to each epoch, with a 50-point overlap (0.2 s step size). A 1024-point fast Fourier transform (FFT) was computed for each segment, resulting in a frequency resolution of approximately 0.25 Hz.

Relative power was calculated for standard frequency bands: delta (δ: 0.5–3.8 Hz), theta (θ: 3.9–7.8 Hz), alpha (α: 8–12.6 Hz), and beta (β: 12.7–30 Hz). Relative power values from all segments were averaged to obtain a single spectral estimate for each epoch and participant. Artifact-contaminated epochs (amplitude exceeding ± 100 µV) were excluded during EEG preprocessing before min–max normalization. No additional extreme outliers were observed in the cleaned relative power data.Therefore, the potential influence of outliers on min–max normalization was minimized. To reduce inter-individual variability in absolute EEG amplitude and emphasize relative spectral patterns, relative power values were normalized for each participant using the following min–max formula:

$$Normalized\;value = 2 \times (X - X_{{\min }} )/(X_{{\max }} - X_{{\min }} ) - 1,$$

where X represents the raw relative power of a given epoch, and X_min and X_max represent the minimum and maximum relative power values across all epochs for that participant. This transformation (range: −1 to + 1) reduces between-subject variability while preserving within-subject spectral characteristics^37^.

### Statistical analysis

Statistical analyses were performed using SPSS 21.0 (IBM Corporation, Armonk, NY, USA). Continuous variables were compared between groups using independent-samples t-tests, while categorical variables were analyzed with χ² tests. For comparisons of spectral power (δ, θ, α, β bands and θ/α ratio) across five brain regions, p-values were adjusted using the Benjamini–Hochberg false discovery rate (FDR) procedure (4 frequency bands × 5 regions + θ/α ratio × 5 regions = 25 comparisons), with significance defined as q < 0.05. For ROC analyses, FDR correction was applied separately within each frequency band (across 5 regions per band). For all significant comparisons, Cohen’s d effect sizes and their 95% confidence intervals (CIs) are reported in the Results section. ROC curve analyses were used to evaluate the diagnostic utility of EEG measures that showed significant group differences. Optimal cut-off values were identified by maximizing Youden’s index. A two-tailed p-value < 0.05 (or q < 0.05 after FDR correction) was considered statistically significant.

## Results

### Demographic and clinical characteristics

No significant differences emerged between the aMCI and NC groups regarding age, gender, education, BMI, hypertension, diabetes, smoking, drinking, ADL, HAMA, HAMD, and HIS scores (all *P* > 0.05). However, MMSE and MoCA scores were significantly lower in the aMCI group compared to the NC group (*P* < 0.05, Table 1).

**Table 1:** Demographic and clinical characteristics of participants.

	aMCI(*n* = 24)	NC(*n* = 24)	*P*-value
Age(year)	65.54 ± 6.82	61.38 ± 8.51	0.07
Male(n,%)	7,29.2%	10,41.7%	0.37
Disease duration(year)	2.83 ± 1.83	-	-
Education(year)	12.00 ± 3.11	12.29 ± 3.61	0.77
BMI(kg/m^2^)	23.25 ± 2.73	24.67 ± 2.49	0.07
Hypertension(%)	10,41.7%	15,62.5%	0.15
Diabetes(n,%)	4,16.7%	8,33.3%	0.18
Smoking(n,%)	3,12.5%	4,16.7%	0.68
Drinking(n,%)	2,8.3%	7,29.2%	0.06
Family history of dementia(n,%)	1,4.2%	6,25.0%	0.07
MMSE	25.88 ± 2.91	28.08 ± 1.34	0.02*
MoCA	24.00 ± 3.15	26.21 ± 1.81	0.01*
ADL	74.58 ± 3.35	73.17 ± 2.22	0.91
HAMA	3.17 ± 2.37	3.21 ± 2.43	0.95
HAMD	5.83 ± 4.27	5.04 ± 4.27	0.49
HIS	0.67 ± 0.64	0.67 ± 0.57	0.68

*p < 0.05.

### EEG abnormalities in the two groups

EEG interpretation can vary based on examiners’ experience, analytical skills, and diagnostic criteria. To minimize bias, two experienced EEG specialists independently assessed all EEG recordings. EEG background activity was graded into five categories according to criteria from Clinical EEG by Liu Xiaoyan: normal, borderline, mildly abnormal, moderately abnormal, and severely abnormal. EEG severity distributions in the two groups are presented in Table 2.

**Table 2:** EEG severity in aMCI and NC groups.

Normal(*n*,%)	aMCI(*n* = 24)	NC(*n* = 24)	*P*-value
	7, 29.2%	8, 33.3%	0.76
Borderline(n,%)	11, 45.8%	13, 54.2%	0.56
Mildly abnormal(n,%)	6, 25.0%	3, 12.5%	0.27
Moderately abnormal(n,%)	0	0	
Severely abnormal(n,%)	0	0	

### Relative spectral power in δ, θ, α, β bands and θ/α ratio between aMCI and NC groups

**Fig. 1:**
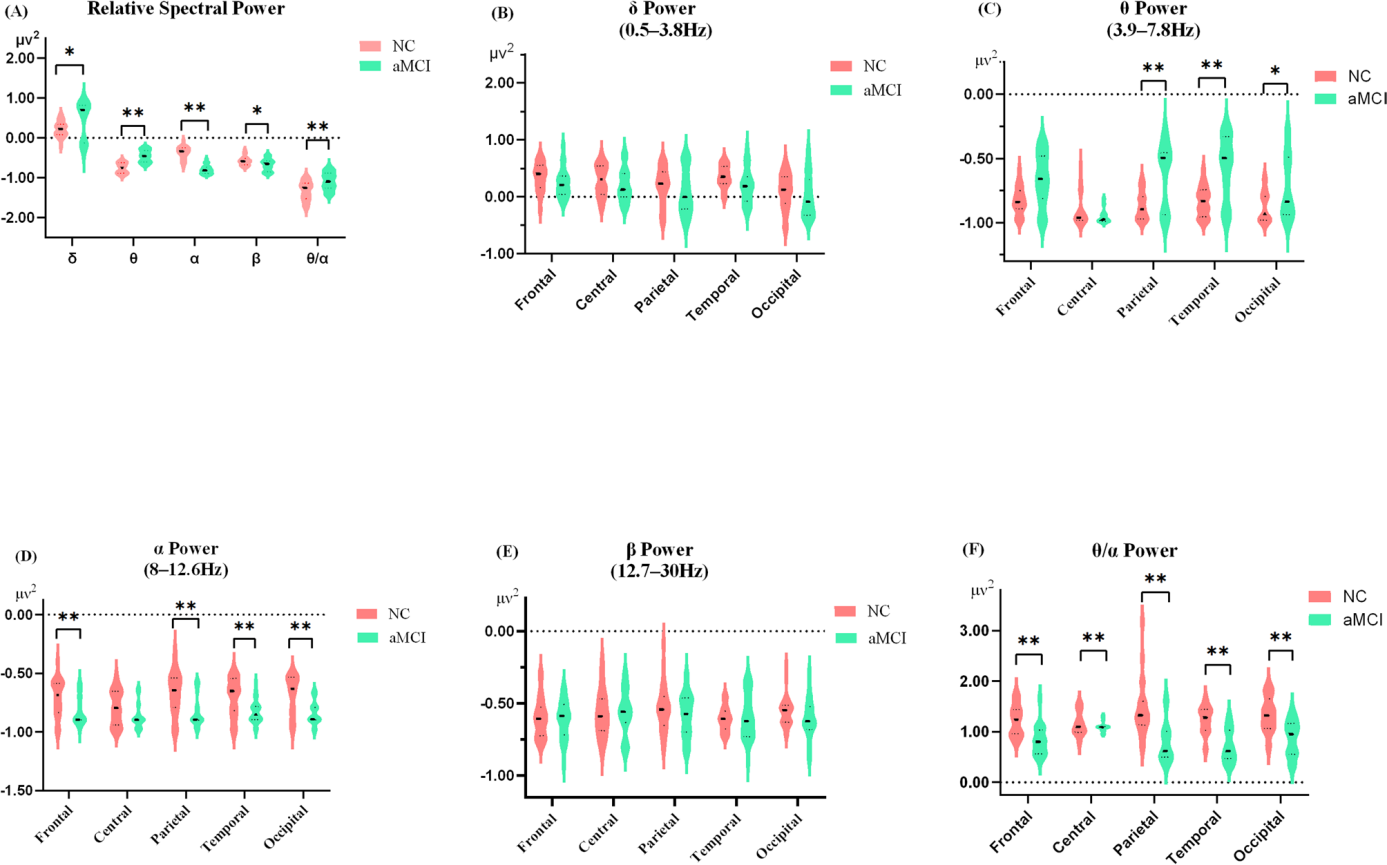
(**A**) Group comparisons of relative spectral power in δ, θ, α, β bands and θ/α ratio (mean ± SEM). (**B**-**F**) Topographic maps showing differences in relative spectral power across frequency bands between aMCI and NC groups. *q < 0.05, **q < 0.001 (FDR-corrected).

Significant group differences in relative spectral power and θ/α ratio were observed at the whole-brain level and specific regions (Fig. 1). Reported p-values were adjusted using the Benjamini–Hochberg FDR method (q < 0.05). Cohen’s d effect sizes with 95% CIs were calculated to quantify these differences.

Delta (δ) Band: The aMCI group exhibited significantly higher relative δ power than the NC group at the whole-brain level (t = -2.196, *p* = 0.036, d = -0.63, 95% CI [-1.21, -0.05]). However, no regional comparisons reached statistical significance after FDR correction (frontal: q = 0.18; central: q = 0.29; parietal: q = 0.29; temporal: q = 0.15; occipital: q = 0.29) (Fig. 1B).

Theta (θ) Band: Relative θ power was significantly elevated in the aMCI group at the whole-brain level (t = -7.340, *p* < 0.001, d = -2.14, 95% CI [-2.84, -1.42]). Regional analyses revealed significantly higher θ power in the aMCI group within the parietal (t = -4.43, q < 0.001, d = -1.28, 95% CI [-1.90, -0.65]), temporal (t = -4.70, q < 0.001, d = -1.36, 95% CI [-1.98, -0.72]), and occipital regions (t = -2.70, q = 0.011, d = -0.78, 95% CI [-1.40, -0.16]). The frontal (q = 0.08) and central (q = 0.12) regions showed trends towards significance but did not survive FDR correction (Fig. 1C).

Alpha (α) Band: Relative α power was significantly lower in the aMCI group at the whole-brain level (t = 10.938, *p* < 0.001, d = 3.16, 95% CI [2.29, 4.01]). This reduction was widespread, evident in the frontal (t = 2.90, q = 0.006, d = 0.84, 95% CI [0.24, 1.43]), parietal (t = 3.67, q < 0.001, d = 1.06, 95% CI [0.45, 1.67]), temporal (t = 3.24, q = 0.003, d = 0.93, 95% CI [0.33, 1.53]), and occipital (t = 4.19, q < 0.001, d = 1.21, 95% CI [0.59, 1.82]) regions. The central region did not remain significant after FDR correction (q = 0.11) (Fig. 1D).

Beta (β) Band: At the whole-brain level, relative β power was also significantly lower in the aMCI group (t = 2.425, *p* = 0.028, d = 0.70, 95% CI [0.11, 1.28]). However, none of the regional comparisons reached statistical significance after FDR correction (all q > 0.05), consistent with the modest global effect size (d = 0.70) (Fig. 1E).

Theta/Alpha (θ/α) Ratio: The θ/α ratio was significantly higher in the aMCI group at the whole-brain level (t = -11.52, *p* < 0.001, d = -3.33, 95% CI [-4.33, -2.33]). Regionally, elevated ratios were confirmed in the frontal (t = -7.93, q < 0.001, d = -2.30, 95% CI [-3.02, -1.55]), central (t = -4.07, q < 0.001, d = -1.18, 95% CI [-1.79, -0.56]), parietal (t = -7.12, q < 0.001, d = -2.29, 95% CI [-2.95, -1.49]), temporal (t = -9.20, q < 0.001, d = -2.66, 95% CI [-3.43, -1.81]), and occipital (t = -10.88, q < 0.001, d = -3.14, 95% CI [-3.99, -2.28]) regions (Fig. 1F).

### ROC analysis of EEG measures for distinguishing aMCI from NC

ROC curve analysis evaluated the diagnostic utility of relative spectral power and θ/α ratio across five brain regions. To control for multiple comparisons, p-values from AUC significance tests (compared to AUC = 0.5) were adjusted using the Benjamini-Hochberg FDR procedure within each frequency band (q < 0.05). The area under the curve (AUC), 95% CIs, optimal cut-off values, sensitivity, specificity, and Youden’s index for each region are presented in Table 3.

**Fig. 2:**
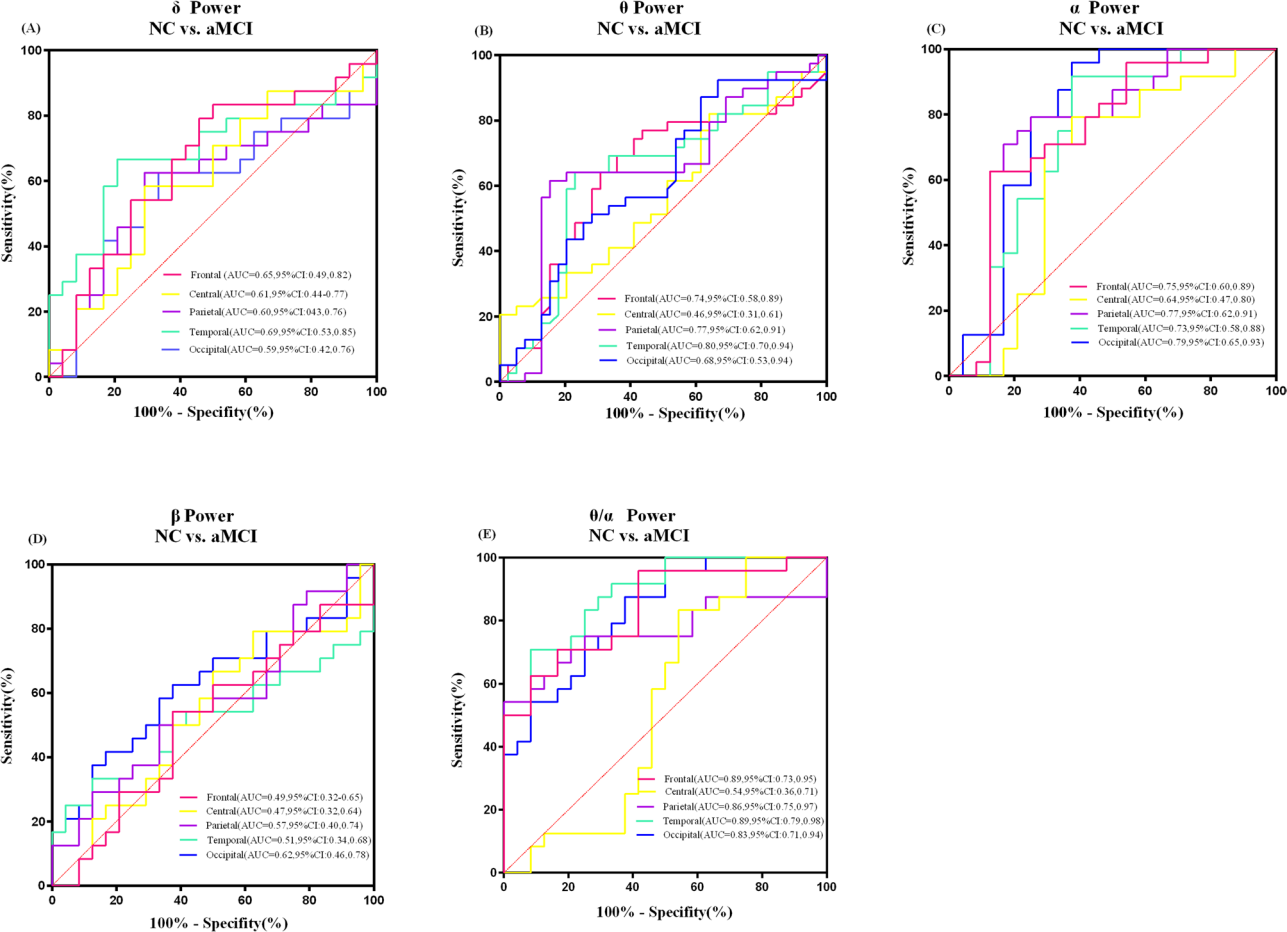
ROC curves for (**A**) δ power, (**B**) θ power, (**C**) α power, (**D**) β power, and (**E**) θ/α ratio across five brain regions. Shaded areas indicate 95% confidence intervals. Curves are color-coded by brain region (frontal: red, central: blue, parietal: green, temporal: orange, occipital: purple) for clarity. *q < 0.05, **q < 0.001 (FDR-corrected).

Delta (δ) Band: After FDR correction, δ power did not demonstrate significant discriminative ability in any of the five brain regions (all q > 0.05) (Fig. 2A).

Theta (θ) Band: θ power showed significant discriminative ability in the parietal (AUC = 0.77, 95% CI [0.62, 0.91], q = 0.01), temporal (AUC = 0.80, 95% CI [0.70, 0.94], q < 0.001), and occipital (AUC = 0.68, 95% CI [0.53, 0.94], q = 0.01) regions. The frontal region displayed a trend towards significance (AUC = 0.74, q = 0.08) but did not remain significant after correction, while the central region showed limited discriminative power (AUC = 0.46, 95% CI [0.31, 0.61], q = 0.12), possibly reflecting region-specific variability in early AD (Fig. 2B).

Alpha (α) Band: α power demonstrated significant discriminative ability in all regions except the central area: frontal (AUC = 0.75, 95% CI [0.60, 0.89], q = 0.01), parietal (AUC = 0.77, 95% CI [0.62, 0.91], q = 0.001), temporal (AUC = 0.73, 95% CI [0.58, 0.88], q = 0.01), and occipital (AUC = 0.79, 95% CI [0.65, 0.93], q = 0.001) (Fig. 2C).

Beta (β) Band: β power did not show significant discriminative ability in any brain region after FDR correction (all q > 0.05) (Fig. 2D).

Theta/Alpha (θ/α) Ratio: The θ/α ratio exhibited strong discriminative performance, remaining significant after correction in the frontal (AUC = 0.89, 95% CI [0.73, 0.95], q < 0.001), parietal (AUC = 0.86, 95% CI [0.75, 0.97], q < 0.001), temporal (AUC = 0.89, 95% CI [0.79, 0.98], q < 0.001), and occipital (AUC = 0.83, 95% CI [0.71, 0.94], q < 0.001) regions. Notably, the central region showed no significant discriminative ability (AUC = 0.54, q = 0.650), indicating that central EEG spectral patterns may not be sensitive to amyloid-related pathology in aMCI. Conversely, frontal, parietal, temporal, and occipital regions demonstrated robust diagnostic performance (AUC range: 0.83–0.89), with the temporal region showing the highest AUC (0.89) (Fig. 2E).

**Table 3:** ROC analysis performance of spectral power and θ/α ratio for distinguishing aMCI from NC across brain regions.

Band	Region	AUC	95% CI	q-value	Cut-off	Sensitivity	Specificity	Youden’s Index
δ	Frontal	0.65	[0.49,0.82]	0.18	0.39	0.79	0.54	0.33
	Central	0.61	[0.44,0.77]	0.29	0.16	0.58	0.71	0.29
	Parietal	0.60	[0.43,0.76]	0.29	0.14	0.63	0.71	0.33
	Temporal	0.69	[0.53,0.85]	0.15	0.22	0.67	0.79	0.46
	Occipital	0.59	[0.42,0.76]	0.29	0.04	0.63	0.67	0.29
θ	Frontal	0.74	[0.58,0.89]	0.08	−0.74	0.79	0.66	0.46
	Central	0.46	[0.31,0.61]	0.12	−0.98	0.04	0.63	0.33
	Parietal	0.77	[0.62,0.91]	0.01*	−0.69	0.96	0.67	0.63
	Temporal	0.80	[0.70,0.94]	< 0.001**	−0.64	0.96	0.67	0.63
	Occipital	0.68	[0.53,0.94]	0.01*	−0.96	0.50	0.92	0.42
α	Frontal	0.75	[0.60,0.89]	0.01*	−0.89	0.58	0.88	0.46
	Central	0.64	[0.47,0.80]	0.11	−0.85	0.79	0.63	0.42
	Parietal	0.77	[0.62,0.91]	0.001**	−0.80	0.75	0.79	0.54
	Temporal	0.73	[0.58,0.88]	0.01*	−0.69	0.92	0.63	0.54
	Occipital	0.79	[0.65,0.93]	0.001**	−0.66	0.96	0.63	0.58
β	Frontal	0.49	[0.32,0.65]	0.89	−0.59	0.46	0.38	0.17
	Central	0.47	[0.32,0.64]	0.89	−0.59	0.33	0.50	0.17
	Parietal	0.57	[0.40,0.74]	0.89	−0.57	0.54	0.63	0.17
	Temporal	0.51	[0.34,0.68]	0.89	−0.70	0.33	0.88	0.17
	Occipital	0.62	[0.46,0.78]	0.18	−0.62	0.58	0.67	0.25
θ/α	Frontal	0.89	[0.73,0.95]	< 0.001**	0.08	0.63	0.92	0.52
	Central	0.54	[0.36,0.71]	0.65	1.12	0.83	0.46	0.29
	Parietal	0.86	[0.75,0.97]	< 0.001**	1.11	0.88	0.79	0.67
	Temporal	0.89	[0.79,0.98]	< 0.001**	0.93	0.71	0.92	0.63
	Occipital	0.83	[0.71,0.94]	< 0.001**	1.25	0.88	0.63	0.50

AUC, area under the curve; CI, confidence interval. All p-values were obtained from AUC significance tests (H_0_: AUC = 0.5) and were adjusted for multiple comparisons within each frequency band using the Benjamini-Hochberg FDR procedure (reported as q-values). Cut-off values were derived from min-max normalized data (range:−1 to + 1). *q < 0.05; **q < 0.001.

## Discussion

This study examined resting-state EEG spectral patterns in patients with amyloid-positive aMCI, rigorously identified by positive PiB-PET imaging. Compared to age-matched cognitively NC, the aMCI group showed prominent EEG slowing, characterized by increased delta and theta power along with decreased alpha and beta power^38^. A key finding was the superior discriminative power of the theta/alpha ratio, particularly in posterior regions, achieving high specificity and AUC values in ROC analyses.

### Interpretation of spectral slowing and pathophysiological correlates

The observed generalized EEG slowing aligns with the established electrophysiological profile of AD and its prodromal stages^39^. This slowing is widely viewed as a marker of impaired neuronal integrity and disrupted cortical network synchronization. The regional specificity observed, with increased theta power prominent in temporoparietal-occipital areas and reduced alpha power primarily in occipital regions, corresponds closely with the topography of early AD pathology. Elevated theta activity, especially in temporal regions, may reflect early dysfunction in medial temporal structures critical for memory^40^. Conversely, reductions in posterior alpha rhythms, a hallmark of resting-state EEG, are consistent with functional neuroimaging findings of hypometabolism and disrupted connectivity within the posterior hubs of the default mode network, regions vulnerable to early AD pathology^41^.

### The theta/alpha ratio: a composite biomarker of network dysfunction

The θ/α power ratio proved more effective in distinguishing groups than individual frequency bands. Its strength likely stems from its composite nature, simultaneously capturing increases in pathological slow-wave activity and decreases in normal fast-wave activity. Therefore, this ratio may offer a sensitive summary measure of the progressive decline in cortical network communication efficiency associated with neurodegeneration^42^.

### Contribution and context within the literature

It is important to contextualize our findings within existing research. EEG slowing in MCI and AD is a well-established phenomenon. The primary contribution of this study is not the initial description of EEG slowing, but rather the provision of detailed, region-specific quantitative data and comprehensive diagnostic metrics (including sensitivity, specificity, and confidence intervals) from a biologically homogeneous cohort of PiB-PET-confirmed, amyloid-positive aMCI patients. This approach strengthens the interpretation of EEG alterations by directly associating them with Alzheimer’s pathology at a symptomatic, pre-dementia stage^43,44^.

### Limitations and future directions

Several limitations must be acknowledged, guiding both interpretation and future research directions. The modest sample size, although sufficient for detecting large effects, limits the stability of some performance metrics, as reflected by wide confidence intervals, and reduces generalizability. Future studies should incorporate larger samples to accurately quantify effect sizes of EEG differences between groups. The use of participant-wise min-max normalization, selected to control for global power differences, is a non-standard method. This may limit comparability with studies using traditional relative power metrics. In addition, although artifact exclusion reduced outlier effects, participant-wise min–max normalization may still be sensitive to extreme values. Future studies may apply more robust normalization strategies such as percentile-based scaling, to further mitigate this limitation. Additionally, aggregating electrodes into broad regions and employing only an eyes-closed condition limits spatial resolution and exploration of state-dependent brain dynamics. The cross-sectional design prevents conclusions regarding the predictive value of EEG metrics for dementia conversion. Moreover, while potential neurobiological correlates (e.g., cholinergic dysfunction, network disconnection) were discussed, these remain hypothetical as direct biomarkers (e.g., cholinergic PET, CSF tau) were not assessed^45^. The diagnostic cut-off values presented are exploratory, derived from this specific cohort. Therefore, their clinical utility requires rigorous validation in larger, independent, and preferably multicenter prospective cohorts^46^.

Future research should prioritize longitudinal studies to evaluate the predictive utility of EEG spectral measures, particularly the theta/alpha ratio, for clinical progression. Integrating quantitative EEG with other biomarkers (e.g., structural and functional MRI, CSF, tau-PET) within multimodal frameworks will be essential to clarify the neurobiological substrates underlying EEG slowing. Such approaches will aid in developing robust, stage-specific diagnostic and prognostic models for AD^47^.

## Conclusion

In conclusion, this study demonstrates that patients with biomarker-confirmed (Aβ+) aMCI exhibit a distinct and quantifiable pattern of resting-state EEG slowing. The theta/alpha ratio, capturing both slow- and fast-wave changes, shows promise for distinguishing aMCI from normal aging. These results provide further proof-of-concept evidence supporting quantitative EEG as a cost-effective and non-invasive adjunct tool for the early identification of individuals on the Alzheimer’s continuum. However, the exploratory nature of these findings and the noted limitations highlight the need for validation in larger, independent studies. This work establishes an essential foundation for future investigations integrating electrophysiological biomarkers into multimodal diagnostic frameworks for AD.

## Data Availability

The original contributions presented in the study are included in the article/supplementary material, further inquiries can be directed to the corresponding authors.

## Abbreviations

MCI: Mild cognitive impairment
aMCI: Amnestic mild cognitive impairment
AD: Alzheimer’s disease
Aβ: Amyloid-beta
PiB-PET: Pittsburgh compound B positron emission tomography
CSF: Cerebrospinal fluid
FFT: Fast Fourier transform
EEG: Electroencephalography
MMSE: Mini-Mental State Examination
MRI: Magnetic resonance imaging
MoCA: Montreal Cognitive Assessment
NIA-AA: National Institute on Aging-Alzheimer’s Association
ADL: Activities of daily living
CDR: Clinical Dementia Rating
HAMD: Hamilton Depression Rating Scale
HIS: Hachinski Ischemic Scale
CAR: Common average reference
FDR: False discovery rate
ROC: Receiver operating characteristic
